# Nutrition Security During Cancer: A Qualitative Investigation Among Patients With Cancer on Active Treatment From an Area of Persistent Poverty

**DOI:** 10.1002/cnr2.70141

**Published:** 2025-02-13

**Authors:** Margaret Raber, Brad Love, Maria Vazquez, Charulata Ghosh, Ruth Rechis, Katherine Oestman, Thy Ho‐Pham, Denise LaRue, Michael T. Walsh, Darya Kizub, Hilary Ma, Karen Basen‐Engquist

**Affiliations:** ^1^ Department of Health Disparities Research The University of Texas MD Anderson Cancer Center Houston Texas USA; ^2^ The University of Texas at Austin, Moody College of Communication Austin Texas USA; ^3^ The University of Texas MD Anderson Cancer Center, Cancer Prevention and Control Platform Houston Texas USA; ^4^ Harris Health System, Community Health and Wellness Bellaire Texas USA

**Keywords:** diet, eating behaviors, food insecurity, nutrition, qualitative, safety net

## Abstract

**Objective:**

The objective of this paper was to qualitatively explore the eating habits and experience of nutrition security during cancer treatment among patients from an area of persistent poverty being treated in a safety net hospital oncology program.

**Methods:**

Eleven in‐depth interviews were conducted with current individuals with cancer who were (1) undergoing active cancer treatment at LBJ Hospital, (2) over 18 years old, (3) English speaking, and (4) residing in an Acres Homes zip code. Reflexive thematic analysis was undertaken by four members of the study team, which brought together diverse expertise in health disparities, nutrition, food culture, and health communication.

**Results:**

Four main themes emerged from the data, including (1) food beliefs and eating behaviors in the context of cancer, (2) social and economic influences on food selection, procurement, and preparation, (3) lived experience of resiliency and coping with limited resources, and (4) the role of relationships (including social support, family, medical teams) in diet and food choice.

**Conclusion:**

Findings from this study begin to fill a gap in knowledge regarding the complexities of managing nutritional needs among patients residing in an area of persistent poverty, which can inform the development of future health systems and community‐based resources for those negotiating both cancer and chronically limited resources.

## 1 Background

A cancer diagnosis can impact diet through therapeutics that influence appetite and taste receptors [1], physical symptoms that reduce one's ability to source and prepare food [2], and the treatment fees, transportation to care, and work disruptions that impact household food budgets [3]. Nutrition security, an expansion of food security defined as consistent and equitable access to healthy, safe, affordable foods essential to optimal health and well‐being, is an important social determinant of health for individuals with cancer given their increased risk for poor diet and related health outcomes [4]. Food insecurity among individuals with cancer has been linked to treatment‐related financial distress, which can contribute to poor adherence to treatment and delaying of care due to financial concerns, as well as reduced quality of life and nutritional health [5]. Poor nutrition during cancer treatment portends worse mortality and morbidity outcomes [6, 7], may exacerbate complications from diet‐related comorbidities and treatment sequelae [8, 9], and increases the risk for certain subsequent cancers [10, 11, 12].

Existing research on food security in cancer populations has mainly focused on acute food insecurity brought on by the cost and disruption of cancer care [5, 13]; however, patients with chronically limited access to healthy food may have unique experiences. Given that food access is influenced by both geography and socioeconomics, examining the experiences of diet and nutrition security among individuals with both food insecurity and cancer who reside in areas of persistent poverty (those who have maintained poverty rates over 20% for at least the past 30 years) [14] will elucidate the lived experience of this population and inform future nutrition security efforts.

Oncology programs in safety net settings offer care to medically underserved populations and report high levels of food insecurity among patients [15, 16, 17, 18]. Like other underserved populations, however, it is likely that safety net clients residing in areas of persistent poverty have developed unique foodways (the structures of food procurement, selection, preparation, and consumption influenced by multifaceted sociocultural factors) to navigate low‐resource environments and limited food access [19, 20]. The dietary habits and nutrition security experiences of these patients have not been well studied, limiting the development and adaptation of interventions for safety net oncology settings. The objective of this research was to explore the eating habits and experience of nutrition security during cancer treatment among individuals from an area of persistent poverty being treated in a safety net hospital oncology program.

## 2 Methods

### 2.1 Setting

This qualitative, phenomenological study was conducted with individuals with cancer, who resided in the Acres Homes area of Houston, Texas, and were receiving active treatment from the Harris Health Lyndon B. Johnson Hospital (LBJ) Oncology Program. Harris Health is a large safety net hospital system, with numerous primary care clinics throughout the Houston area and two acute care hospitals. Acres Homes is a historically Black neighborhood and includes several persistent poverty census tracts. The area is considered a food desert, with two small grocery stores serving over 57 000 residents.

### 2.2 Participants and Recruitment

In‐depth interviews were conducted with a convenience sample of current patients with cancer who were (1) undergoing active cancer treatment at LBJ Hospital, (2) over 18 years old, (3) English speaking, and (4) residing in an Acres Homes zip code. Individuals were identified through LBJ medical records and contacted via phone by staff in the LBJ Cancer Resource Center. LBJ staff briefly explained the study and requested permission to forward contact information to the study coordinator if patients were interested in being interviewed. The study coordinator then called all interested participants to further explain the study, answer any questions, and schedule interviews. All participants were offered a $20 gift card for their time.

### 2.3 Data Collection

Interviews were conducted by research staff with experience in conducting interviews in diverse populations. All staff completed a qualitative research training course and conducted practice interviews prior to working with study participants. Interviews were up to 1 h in length and conducted over the phone using a semistructured interview guide (Supplement S1), which included questions about perceptions of food access, nutrition and wellness issues, personal definitions of healthy eating, as well as facilitators/barriers to achieving health goals. Interviews were recorded and transcribed verbatim using a professional transcription service. Basic demographics, including age, race/ethnicity, and cancer site, were extracted from the medical record.

### 2.4 Statistical Methods: Qualitative Analysis

Reflexive thematic analysis was undertaken by four members of the study team, which brought together diverse expertise in health disparities, nutrition, food culture, and health communication. The analysis followed a five‐step process, informed by Braun and Clarke [21, 22]. Step one included all team members reading through and listening to the interviews and taking independent notes on overall impressions and main emergent ideas. The analysis team then convened to reflect on the interviews, compare notes, discuss overall thoughts, and outline potential global themes. In the second step, the team reconvened and identified four broad codes, working definitions of the codes and related rules. Three interviews were coded by the entire team as a group over the course of 2 weeks, during which time the code definitions were merged, expanded, and/or clarified until the codebook was finalized and no new codes or themes emerged. All 11 interviews were then independently coded by two members of the analysis team. Coding was compared for consistency between the coders, and discrepancies were discussed by the entire analysis team. As needed, codes were further collapsed, separated, and/or refined as required. Themes were thus finalized, defined, and named by the analysis team, and representative quotes were identified in a group setting. Quotes were identified based on their representativeness of the themes and alignment with patterns in the broader data set, as well as targeted to include a diversity of respondents.

## 3 Results

### 3.1 Participants

Interviews were conducted with 11 patients with cancer undergoing active treatment at LBJ. Almost all (*n* = 10) were African American and one was Hispanic White. All but one participant were aged 50–69 years, while one was aged 40–49 years. Seven participants were female. Cancer diagnoses included breast (*n* = 4), colon (*n* = 3), and other (*n* = 4).

### 3.2 Thematic Analysis

Four themes emerged from the interviews (Table 1): (1) Attitudes and beliefs around food and diet in the context of cancer, (2) complex medical and social situations that influence food selection, procurement, and preparation, (3) lived experience of resiliency and coping with limited resources, and (4) the role of relationships in diet and food choice.

**TABLE 1:** Overarching themes and select top‐level codes.

Theme	Level 1 codes	Level 2 codes
Attitudes and beliefs about food and diet in the context of cancer	Current dietary practices	Healthy
		Unhealthy
	Influence of cancer	Treatment effects
		Symptoms
	Perceptions of the role of diet in health	Motivation for diet change
		Willingness to change
Complex medical and social situations influencing food selection, procurement, and preparation	Social/communication influencers	Information seeking
		Information sharing
	Social Determinants of Health	Food security
		Transportation
		Financial Health
	Food availability	Unhealthy food selections
		Food Prices
Lived experience of resiliency and coping with limited resources	Coping	“Just deal” attitude
		Resilience
		Sales/Lower prices
	Acknowledgement of limitations	Limited resources
		Lack of social support
Roles social support structures and medical teams play in diet and food choice	Medical team information sharing	Diet/nutrition counseling
		Referrals
	Social support	Caregivers
		Professional aides
		Family and Friends
		Neighborhood
	Perceived isolation	
	Perception as a burden	On family
		On care team

### 3.3 Attitudes and Beliefs About Food and Diet in the Context of Cancer

Patients discussed the foods they chose to purchase, prepare, and consume in the context of the cancer experience, including treatment effects, nutritional value, and quality. When discussing current dietary practices, many were insightful about the food choices that they were making.

I think eating greens and fish three times a week and making sure that I'm not eating a lot of fat, 'cause I can't stand that grease. It helps the bloating and all the issues that make you feel fatigued. Female, 56, African‐American.

Not all respondents perceived their diets as healthy. Those who struggled with less healthy eating appeared aware of the situation and sometimes derided themselves for a lack of self‐control over their diet.

I know I got diabetes, but I've got to have that sugar too. I've got to have it. It's bad because it's like I got to have cake or cookies, something of that nature, every day. … I mean, it's tired. I mean, it's pitiful. Lately I've been trying to wean myself off of the cereal and the cake, but it's a hard job. Female, 56, African American.

Cancer treatment effects influenced how and what participants ate. Chemotherapy and radiation were noted as having a detrimental effect on patients' ability to prepare or eat meals, and food choice was subject to variability in appetite related to treatment.

Well after the chemo treatments that I go to, after every chemo treatment after day two and day three my body kinda starts shutting down and I started gaining weight. I really can't do nothing. Male, 59, African American.

The first couple of days are my worst days. I mean, the first day I get it and a couple of days after then, my arm be like froze. Like if I cook, it's gonna hurt. I'm trying to learn how to deal with that more. If I have to keep on doing this I've got to learn how to try to deal with it. Female, 56, African American.

Many noted diet changes in a positive light, with cancer acting as a catalyst for healthier eating.

Well, for me when I was goin’ through it [cancer treatment], I didn't really make a lot of negative changes ’cause how my meals was pretty bad anyway, my diet, my exercise and everything. To me, it was a positive. ’Cause I started doin’ things I needed to do except for maybe somedays I didn't feel like goin’ or doin’ it or stuff like that. The energy, I had to make myself get up and do it. Still, that wasn't a negative to me because I was doin’ it for me. Female, 61, African American.

One thing I done, I changed my eating habits. I used to love, before I found out I had cancer, I used to love to go to a place called Timmy Chan's, get me some—it might be every other day. I'm going to get me a chicken wing with some French fries… I had to get away from the fried foods. Male, 54, African American.

Yeah, it [cancer] started me eating more healthy food than normal food that I would eat. Pretty much like fresh vegetables and fruits. Male, 59, African American.

Some patients also described wanting more information to inform their dietary practices in the context of cancer. This information could come through their healthcare teams via their family/friends or on their own.

My son has been pretty much looking up—about what food is good for cancer and what food is not good for cancer. Because I don't wanna be feeding it, and it's growing. I wanna help them destroy it. I don't wanna be—I wanna know what I should and should not be eating, not just talk about general nutrition like you do every day with a regular person. Female, 56, African American.

While patients appreciated the importance of diet during cancer treatment, desired changes were not always practical. The complexities of medical and social situations faced by interviewees were evident in how they described the many factors that influenced their food purchasing and preparation, as well as in how they viewed the relationships between previous food choices, treatment effectiveness, and considerations of life after diagnosis.

### 3.4 Complex Medical and Social Situations Influencing Food Selection, Procurement, and Preparation

Social, healthcare, and economic realities were noted as barriers to healthy eating during cancer. This included the cost of ingredients and the time commitment to prepare healthy meals.

It's so expensive. I've always rather clean up the biggest mess than have to sit there and cook, and especially pay for it. Female, 63, Hispanic/Latino.

Like the vegetables. I mean, they really went up. Dang. I mean, oh, Lord, they went up. Like some of the meats like chicken, seafood. You know? That's supposed to be healthy for you, but [prices], I mean, skyrocketed. Female, 56, African American.

Family and broader social circles, both in person and online, influenced what types of foods patients ate, and patients described their home food environments in terms of other household members and food norms.

Me and my husband do it together. My son lived here then, and he did more, I guess, fresh vegetables, like squash and stuff, than we did. There were three of us preparin’ it at the time. Now, it's just me and my husband. We cooked together. We planned meals together, but we don't make a big meal every day. Female, 63, Hispanic/Latino

I love my mother. My mother knows how to cook well, so I leave it to her…One thing, my mother, she's a very picky lady. She don't want nobody else cooking nothing for her. She gonna cook it for herself. Male, 54, African American.

She's [sister] a health fanatic. I'm not playing. I'm not playing. That girl had me eating stuff that I found out, I was like, “Huh‐uh. I don't want this.” Female, 56, African American.

Along with the home, the neighborhood food environment was discussed as a major influencer on the patient's diet. Although most participants noted the high availability of food stores and restaurants, this was also interpreted as a negative because of fast food and unhealthy foods dominating the local foodscape.

This is a junk‐food neighborhood, honey. All of the junk food you can dream about eating, you can eat it. Female, 56, African American.

### 3.5 Lived Experience of Resiliency and Coping With Limited Resources

Participants shared several insights that illuminated their unique foodways and overall approach to the cancer experience. Many patients emphasized the importance of self‐perception, noting how things could be worse and rationalizing less‐than‐ideal scenarios. One patient, in describing a seven‐hour wait to see their physician, noted:

Yeah, I sat there a while, but with the pain I was in, I wasn't fixing to go nowhere. In fact, if I had to sit and wait a little longer, that would've been no problem. Male, 54, African American.

Moving forward was a common theme among participants when describing their experiences of food budgeting. While some mentioned looking for sales and other tactics to reduce food costs, others simply accepted high food costs as a fact to be endured. Several participants described a balancing act between high prices and nutritional needs, with nutritional priorities overcoming wariness regarding food costs.

It can get expensive for your proper food. If you have to eat nuts—I like walnuts, whole grain stuff, yogurt and stuff is good for you, it's mostly more expensive, but you need. Female, 56, African American.

Well, it's not as affordable as regular food, but it's better for ya, but sometimes you gotta break down and buy sometime… If I think it's healthy somethin’, and I want it, my son just buy it. Female, 61, African American.

I don't buy organic stuff, but I know it's pricier than normal stuff, so I could say no. Because regular food is already ridiculous, I'm sure fresh fruits and stuff is ridiculous… That doesn't mean I don't buy it. We bought cherries today. We had watermelon the other day and strawberries… Female, 63, Hispanic/Latino.

There was a limited acknowledgment of the need for financial or social support related to food. Even when patients described financial distress, it was framed as a temporary phase to be overcome.

If you have a little bit of income, it [healthy food] is affordable. If you have a little bit of income. It depends. Sometimes when finances are pretty hard—for example, right now I am not able to work. My husband has to take care of everything in the house. Our finances are not—our finances are not at our best, but we are trying our best. Female, 56, African American.

### 3.6 Roles Social Support Structures and Medical Teams Play in Diet and Food Choice

Through cancer treatment, patients interact with a number of systems and individuals that influence diet. With regard to medical teams, some patients indicated a lack of communication about diet in the context of cancer from their physicians. This was sometimes validated by patients through the perception that the oncology teams were themselves unable to share such information.

They [the medical team] don't know nutrition. They know medicine, and they know how to diagnose, and they know how to do procedures. They don't know nutrition. I have to do that. Male, 46, African American.

Family was the main social support noted by participants, including parents and adult children. Family members often took on food purchasing and preparation, as well as informing themselves of the nutritional needs of individuals with cancer.

Well, with me and my situation, it's just I have a child that can afford to come and take care of me… I wouldn't have been at home by myself. To try to do that all by myself, I couldn't have did all that. Them had to prepare my meds and making sure I eat right, which my son is in medicine himself. He knows what all I needed so I could eat right. He learned about calcium, what I should be doing and what food I should be eating and what food I shouldn't be eating. Female, 56, African American.

Reliance on family varied, sometimes following a trajectory of support during certain parts of treatment and then self‐reliance once able. Reasons for family reliance were usually linked to physical ability, as opposed to financial limitations. Participants highlighted the importance of these familial relationships in their nutritional health.

At the beginning it was different, but now I'm here with my son and my fiancé. They do pretty much everything for me that needs to be done I can't do for myself. Female, 56, African American.

I have this neuropathy sorta bad right now, so I can't really cook what I wanna cook, but my son, he gets in there and do for me. Female, 61, African American.

In relationships with both medical teams and family, there was awareness of the impact of the self on others. This sometimes manifested as a desire to not be a burden, either in financial expense or time/effort. Patients also noted their care as a burden on physicians, with their nutritional health being outside the necessary purview of already‐busy doctors. Some negotiated this issue by taking specific actions taken to reduce the burden on others, or at least their perception of it.

They got a lotta patients to help. They only get to see me for a few minutes. Now, we stick to the diagnosis and the prognosis and scans and stuff, and don't spend a lotta time talkin’ about food. Female, 63, Hispanic/Latino.

Whatever you're going through. You know? You can't put your burden on nobody else. You've got to depend on yourself…I don't ask my family to help me too much, but to get me back and forth to the clinic. Everything else I do on my own, I do for myself. I go to the washeteria for myself; I cook for myself. I don't let it get me down. Female, 56, African American.

Many patients expressed varying levels of isolation. In some cases, this was absolute, with patients rarely leaving their homes.

It changed some time ago. Three or four years ago. It's changed when this COVID thing—I used to go outside, and walk the driveway, and go outside and walk by myself. Then it changed… I don't go anyplace right now. Male, 68, African American.

## 4 Discussion

This qualitative study examined foodways and nutrition security among individuals with cancer from a persistent poverty area being treated at a safety net hospital. The findings offer insights into nutrition security and diet‐related attitudes and beliefs, as well as lived experiences and the role of relationships among patients in a complex sociomedical environment.

Although all participants in this study resided in a food desert, the overwhelming majority felt they had access to a diversity of foods and that acquiring healthy foods was not a matter of location. Several large cross‐sectional studies have linked residence in a food desert with poor diet [23]; however, qualitative studies with noncancer populations reveal a more nuanced picture of the ways that people cope with and adapt to geographic challenges. Aligned with our findings, Alkon et al.'s study of foodways of the urban poor, which interviewed or surveyed more than 580 individuals, revealed that cost was a more significant barrier to healthy eating than lack of knowledge or physical proximity to food outlets [19]. This finding is further supported by MacNell's geoethnographic analysis of (*n* = 100) low‐income mothers residing in food deserts, which found that participants were highly willing to bypass closer stores to reach their preferred markets [24]. In addition to these motivations, participants in the current study all resided in the Acres Homes neighborhood of Houston, Texas, a sprawling metropolis with limited walkability, and little public transport. Thus, many residents may be accustomed to grocery shopping only when they have access to transportation, reducing the impact of distance on their perception of food access.

Participants in our study described prioritizing healthy food purchasing despite being wary of prices. This prioritization seemed to be related to the way participants perceived the importance of diet in relation to their physical well‐being while undergoing cancer therapy, further indicating many described positive changes in diet after a cancer diagnosis. This phenomenon has been noted in other cancer populations, and a recent narrative review identified decreased red/processed meat consumption and increased consumption of fruits and vegetables as common changes after diagnosis [25]. These findings suggest individuals with cancer living in areas of persistent poverty may be amenable to healthy eating interventions that have been successful in other cancer populations.

Among participants, factors linked to dietary changes after diagnosis included support from caregivers, who grocery shopped, cooked healthy meals, or researched the role of food in cancer on their behalf. The role of caregivers has emerged in other studies examining influencers of dietary change in the context of cancer, not specific to persistent poverty. Across several diagnoses, including prostate [26], colorectal [27], and gastrointestinal [28] cancers, caregivers were consistently noted as major influencers on postdiagnosis diet.

Further examination of the way patients negotiated their provider and familial relationships during therapy revealed many instances of reliance, as well as some feelings of burden. Perceptions of burden presented at times as a barrier to seeking family support around financial needs or time commitments, and the same was true at times of not wanting to request dietetic support from clinicians who were perceived as already negotiating many demands. These findings are in line with recent work on the high prevalence and challenging nature of self‐perceived burden among those affected by cancer [29, 30]. High self‐perceived burden can relate to reduced quality of life and increased isolation, as observed in several interviews here, making clear the need for further investigations in this area [31].

### 4.1 Clinical Implications

This study highlights the necessity and opportunity for discussions between health care professionals and patients on nutrition security in the context of safety net oncology care. An examination of the way patients negotiated their provider and familial relationships during therapy revealed many instances of reliance, as well as some feelings of burden. Perceptions of burden presented at times as not wanting to request dietetic support from clinicians who were perceived as already negotiating many demands, factors in line with other studies on the high prevalence of self‐perceived burden among those affected by cancer [29, 30].

Additionally, this study demonstrated the need and potential impact of interventions to enhance nutrition security in safety net settings. Many interviewees described positive changes in diet after a cancer diagnosis. This phenomenon has been noted in other cancer populations, and a recent narrative review identified decreased red/processed meat consumption and increased consumption of fruits and vegetables as common changes after diagnosis [25]. These findings suggest individuals with cancer in a safety net setting may be amenable to healthy eating interventions that have been successful in other cancer populations.

### 4.2 Study Limitations

This study has several strengths, including in‐depth interviews with a hard‐to‐reach population of individuals with cancer residing in persistent poverty areas. A multistep thematic analysis process was undertaken by four experienced researchers with diverse expertise. This study is limited by the small sample size and highly specific population, which limits the generalizability of findings to other safety net centers. Recruitment was not restricted by cancer type, and information about cancer stage or treatment protocol was not collected, limiting conclusions regarding how these factors influence diet. However, 11 interviews are in line with other phenomenological research on specified populations [32], and our coding scheme was stabilized after group coding of 3 interviews, suggesting some level of saturation [33]. Other limitations include the use of audio‐only interviews, which limits our ability to understand nuanced body language during the interviews.

### 4.3 Conclusion

Results from this study, focused on reported experiences from people undergoing active cancer treatment at a safety net hospital, begin to fill a gap in knowledge about the complexities of managing nutritional needs in an area of persistent poverty, which can inform the development of future resources for patients negotiating chronically limited resources.

## Author Contributions

**Margaret Raber, Brad Love, Maria Vazquez, and Charulata Ghosh:** carried out the analysis and original writing of this manuscript. **Ruth Rechis and Katherine Oestman Michael T. Walsh:** provided editing to the paper. **Thy Ho‐Pham, Denise LaRue, Darya Kizub, and Hilary Ma:** provided coordination of patients to the research coordinator for the interviews conducted. **Karen Basen‐Engquist:** received funding for this project and provided supervision to the study. All authors agree to the final version of this manuscript.

## Ethics Statement

This project was approved by the University of Texas MD Anderson Cancer Center Quality Improvement and the Harris Health Quality Improvement Assurance Board.

## Consent

This study conforms to recognized standards of the US Federal Policy for the Protection of Human Subjects. All patients were provided an informed consent document of the study and agreed to participate.

## Conflicts of Interest

The authors declare no conflicts of interest.

## Supporting information


**Data S1.** Supporting Information.

## Data Availability

The data that support the findings of this study are available on request from the corresponding author. The data are not publicly available due to privacy or ethical restrictions.
