# Supplementary material for: Nutrition Security During Cancer: A Qualitative Investigation Among Patients With Cancer on Active Treatment From an Area of Persistent Poverty
Source: Cancer Rep (Hoboken). 2025 Feb 13;8(2):e70141. doi: 10.1002/cnr2.70141 (PMC11825291; doi:10.1002/cnr2.70141)
Supplement: Supplementary file 1 — Data S1. Supporting Information. [file CNR2-8-e70141-s001.docx]

What does healthy eating mean to you?

How has cancer treatment impact your diet?

Who has been preparing food during treatment?

Do you think you have a healthy diet right now?

[IF NO] 4a) What keeps you from eating healthy?

[IF YES] 4a) What helps you to eat healthy?

What kind of stores, markets or restaurants are located near you?

In your opinion, is healthy food affordable?

[IF NO] 6a)Can you give me an example of a healthy food that is too expensive?

[IF YES] 6a)Can you give me an example?

Thinking about your most recent visit, from the moment you walk in, to the moment you walk out, what does the process look like?

Do you and your healthcare provider talk about healthy eating and exercise?

[IF NO] – a) Would you like to talk to them about healthy eating and exercise? What type of information would you like them to provide?

[IF YES]- a) Do they give you recommendations about healthy eating and exercise?

b) What do they tell you?

c) Do you follow their advice?

[IF NO]- c.1) Why not?

d) Do they refer you to any resources?

[IF YES]- d.1) what resources did your provider offer?

d.2) did you use that resources?

e) What other resources do you think would be helpful to you?
